# The Impact of Disinfecting Non-Sterile Disposable Gloves on the Level of Microbiological Contamination in Clinical Practice

**DOI:** 10.3390/microorganisms14020286

**Published:** 2026-01-26

**Authors:** Anna Gajkiewicz, Julia Szymczyk, Sandra Lange, Wioletta Mędrzycka-Dąbrowska

**Affiliations:** 1 Sanitary and Epidemiological Station in Malbork, Juliusza Słowackiego 64, 82-200 Malbork, Poland; gajkiewicz.anna@gmail.com; 2 Department of Anaesthesiology Nursing & Intensive Care, Faculty of Health Sciences, Medical University of Gdansk, Dębinki 7, 80-211 Gdańsk, Poland; szymczykjulia@gumed.edu.pl; 3 Department of Internal and Pediatric Nursing, Medical University of Gdańsk, Dębinki 7, 80-211 Gdańsk, Poland; langa94@gumed.edu.pl

**Keywords:** contamination, microbiological, non-sterile, gloves

## Abstract

Gloves, used in conjunction with hand hygiene, are designed to protect healthcare personnel from direct contact with blood, body fluids, and other potentially infectious materials, which is critical for reducing the transmission of microorganisms. The aim of this systematic review was to analyze available studies on the disinfection of disposable, non-sterile gloves as a method of reducing the risk of microbial contamination in everyday clinical practice. A systematic review was conducted in the fourth quarter of 2025. A total of 317 records were initially retrieved from the five databases (EBSCO, PubMed, Scopus, Web of Science, Ovid). Interventions included alcohol-based hand rubs (ABHR), sodium hypochlorite wipes or solutions, quaternary ammonium wipes, and sporicidal ethanol. Across all studies, glove disinfection consistently reduced bacterial, viral, and spore contamination. Hypochlorite-based agents and sporicidal ethanol demonstrated the highest efficacy against spore-forming organisms such as Clostridioides difficile. Alcohol-based hand rubs were effective against bacteria and enveloped viruses but showed reduced activity against non-enveloped viruses and spores. Conclusions from studies conducted in both laboratory and clinical conditions clearly emphasize the key role of hand hygiene after removing gloves, even when using multiple layers of protection, while also indicating that glove disinfection can be a useful supplement to protection against particularly virulent pathogens (EVD, CDI).

## 1. Introduction

For many decades, hand hygiene and the correct use of disposable gloves have been the cornerstone of infection prevention in healthcare facilities. Gloves, used in conjunction with hand hygiene, are designed to protect healthcare personnel from direct contact with blood, body fluids, and other potentially infectious materials, which is critical for reducing the transmission of microorganisms. The use of both mechanisms by healthcare personnel significantly increases the chances of effective protection against pathogens [1]. Selective use of gloves instead of comprehensive hand hygiene can lead to a false sense of security and a greater risk of contamination of staff hands and the hospital environment. This practice results in an increased risk of infection for both patients and staff, which poses serious clinical, social, and scientific challenges [2]. Given the emergence of multidrug-resistant bacteria (MDRO) in healthcare facilities over the past two decades, hand hygiene and the proper use of gloves have become an absolute necessity [3]. Data from a literature review indicate that the spread of carbapenem-resistant strains, among others, results in an increased number of CPE infections and a high risk of death. The mortality rate for these infections ranges from 30 to 70%, and in bloodstream infections it can exceed 50% [3].

Detailed international guidelines and national epidemiological recommendations on the use of personal protective equipment and hand hygiene determine the quality of healthcare services. Both hand hygiene and the correct use of gloves are procedures that determine the number of hospital infections and thus become a key tool in the prevention of nosocomial infections [2]. In order to standardize the rules for hand hygiene and glove use, the WHO has developed the concept of “5 moments of hand hygiene,” recommendations for the use of alcohol-based disinfectants, and guidelines for the proper use of medical gloves [4]. With regard to the correct use of gloves, it is important to mention the basic rules for their use. First and foremost, they are used to protect against contact with blood and other body fluids and to prevent the transmission of microorganisms between patients and staff. The recommendations clearly state that the same gloves should not be used on more than one patient and that they should be changed when caring for a single patient, e.g., after performing a procedure with a high risk of contamination and before moving to clean areas. It should be emphasized that gloves do not provide complete protection against infection, and washing or disinfecting them remains contrary to established practice. Putting on and taking off disposable gloves is inseparable from hand hygiene. As a rule, personal protective equipment cannot replace basic hygiene procedures, but unfortunately, in clinical practice, gloves are used as an alternative. The use of gloves without washing or disinfecting the hands before putting them on increases the risk of pathogen transmission, contaminates the hands of staff, and contradicts recommendations [1,2,3]. These recommendations consistently exclude the possibility of using non-sterile disposable gloves, pointing to a lack of sufficient scientific evidence regarding the safety of this practice and the risk of damage to the structure of the material under the influence of alcohol or other chemicals [4]. As cited by Verbeek et al., the correct use of full personal protective equipment (PPE), including gloves, as well as proper techniques for putting on and taking off this equipment, are key to reducing the risk of self-contamination and infection among healthcare workers. In situations of epidemics of highly contagious diseases, such as Ebola Virus Disease (EVD), Severe Acute Respiratory Syndrome (SARS), or COVID-19, medical personnel are much more exposed to infection due to contact with infected body fluids. Evidence, albeit of low quality, suggests that double gloving may reduce the risk of contamination compared to single gloving. However, further research is needed on optimal methods of PPE use and effective forms of staff training to increase protocol compliance and work safety [5]. Until the COVID-19 pandemic, medical personnel did not practice disinfecting non-sterile disposable gloves due to the lack of the above recommendations. However, in the face of a new unknown pathogen and a shortage of personal protective equipment in the form of gloves, institutions such as the RKI [6], CDC [7], and ECDC [8] introduced modifications to established practices. Glove disinfection became acceptable in two situations: when performing various activities on a single COVID-19 patient (in accordance with the 5 moments of hand hygiene) and when removing protective clothing when leaving the COVID-19 zone. Following the recommendations, there has been an increase in interest in the disinfection of disposable gloves, which has been reflected not only in clinical practice. Unlike previous reviews on glove reuse, this is the first to focus on disinfection efficacy in reducing microbial load post-single use.

### 1.1. Background

Although current guidelines do not recommend disinfecting disposable non-sterile gloves during patient care, the results of some studies suggest that this practice could be useful in other contexts, for example, as part of a safety strategy when removing gloves (doffing), especially after contact with patients infected with Clostridioides difficile or multidrug-resistant organisms (MDROs). This seems particularly relevant in multi-bed wards equipped with only two sanitary facilities and one isolation room, where medical staff are increasingly faced with the need to combat outbreaks [9]. Previous literature reviews, such as the analysis by Kampf and Lemmen, indicate the significant potential of disinfecting disposable gloves as a complementary strategy for reducing the transmission of microorganisms during multiple medical procedures on the same patient. The results suggest that properly applied glove disinfection does not compromise their integrity and may reduce the risk of infection. At the same time, there is a knowledge gap regarding the microbiological effectiveness of such practices in different clinical settings and their impact on patient and staff safety. Therefore, further high-quality research is needed, especially randomized controlled trials, to clearly assess the effectiveness of glove disinfection and the conditions for its recommendation in everyday clinical practice [10]. A promising study confirming the safety of glove disinfection is the study by Brinbach et al., which showed that repeated use of an alcohol-based preparation on nitrile gloves during simulated procedures does not cause damage (no increase in micropores) and does not affect tactile sensitivity, although users notice an increase in glove stickiness. The conclusions suggest that ABHR glove disinfection does not compromise their integrity or hinder routine medical procedures. However, this study does not answer the question of the microbiological effectiveness of disinfection, but only assesses the integrity of the gloves and manual dexterity after the application of the alcohol-based product [11]. 

In view of the continuing practice of disinfecting gloves after the COVID-19 pandemic and the recommendations appearing in some studies [12,13,14], there is a real need to verify whether the disinfection of disposable, non-sterile gloves is indeed a safe and effective solution. Key questions concern situations in which disinfection may be recommended, the type of agents used, and the conditions necessary to ensure the safety of patients and staff. Questions remain open regarding the effectiveness of disinfecting non-sterile gloves in preventing the transmission of microorganisms during various medical procedures. Attempting to answer these questions may bring potential benefits to clinical practice.

### 1.2. Aim

The aim of this systematic review was to analyze available studies on the disinfection of disposable, non-sterile gloves as a method of reducing the risk of microbial contamination in everyday clinical practice.

As recommended by the Joanna Briggs Institute (JBI) and PRISMA 2020 guidelines, research questions were formulated according to the PICO framework (Population, Intervention, Comparison, Outcomes) [15,16].

The research question was: Does disinfection of non-sterile disposable gloves (I) by healthcare personnel (P) reduce their microbial contamination (O) compared to no disinfection (C)?

## 2. Methods

### 2.1. Study Design

This systematic review was guided by the JBI Systematic Reviews [15], and was conducted following the recommendations of the PRISMA statement [16]. Given the anticipated limited number of eligible studies on this emerging topic, the review was exploratory in nature (Supplementary Materials).

### 2.2. Search Methods

The following databases were searched: EBSCO, PubMed, Scopus, Web of Science, and Ovid. The following keywords were used: (‘health personnel’, ‘healthcare workers’, ‘medical staff’, ‘nurses’, ‘nurse’, ‘doctor’, ‘medical personnel’), gloves (‘gloves’, ‘disposable gloves’, ‘single-use glove’, ‘non-sterile gloves’, ‘protective gloves’), disinfection (‘disinfection’, ‘decontamination’, ‘sanitization’, ‘cleaning’, ‘sterilization’) and microbial contamination (‘microbial contamination’, ‘microbiological contamination’, ‘bacterial contamination’, ‘contamination’), Keywords were entered along with their combinations using AND or OR. The articles found during each search test were limited to studies conducted between 2015–2025. The literature search was done by AG and JS. All publications were analyzed by title and abstract to exclude duplicates and irrelevant entries. Full-text articles were then read and critically evaluated according to the eligibility criteria and PICO framework. All inclusion and exclusion criteria and search strategies applied to the search are presented in Table 1. The final search was carried out on 11 November 2025. The search strategies are presented in Table 2.

### 2.3. Search Outcomes

A total of 317 records were initially retrieved from the five databases. After removing duplicates 259 and screening titles and abstracts,189 records were excluded. 57 full-text studies were assessed for eligibility. The primary reasons for exclusion were: The most common reasons for exclusion of full-text articles were: 1-lack of actual disinfection of non-sterile gloves (n = 38), 2-lack of a control group with no glove disinfection (n = 11), 3-results not related to microbiological contamination of gloves (n = 2). Ultimately, the final analysis included a total of 6 studies.

### 2.4. Data Extraction

Data were extracted by two researchers: AG and JS. The data extraction template has not been piloted prior to its implementation. The following information was extracted: author and year of publication, aim, study group, materials and methods, results, implications for nursing practice.

### 2.5. Quality Assessment

Two researchers independently assessed the quality of the included studies (AG, JS). The JBI Checklist for randomized controlled trials [17] critical appraisal tools were used to assess the quality of the included studies. The results were divided into three categories: high (if ≥80% of the items on the assessment tool received a point), moderate (if ≥65% of the items on the assessment tool received a point), and low (if ≤55% of the items on the assessment tool received a point). A point was awarded only if the answer to the question in the tool was “yes.” Any discrepancies were resolved through discussion until consensus was reached or consulted with supervisors (WM-D, SL), thus minimizing selection bias. The results of the critical appraisal of quality are presented in Table 3.

### 2.6. Data Synthesis

Narrative synthesis was used to analyze the results of the studies included in this review [22]. According to the model developed by Popay et al., the synthesis process consisted of four stages. (1) First, the approach used in each intervention (glove disinfection method) and target group (healthcare personnel using non-sterile gloves in laboratory and hospital settings) were identified, and the results of the studies included in the review were preliminarily synthesized. (2) The relationships within and between individual studies were examined, and (3) the robustness of the synthesis was assessed. (4) The effectiveness of the studies was described based on the microbiological results of the gloves tested (whether glove disinfection significantly reduced their contamination).

### 2.7. Ethical Approval

Since this study was a systematic review, ethical approval was not required.

## 3. Results

### 3.1. Study Selection

**Figure 1:**
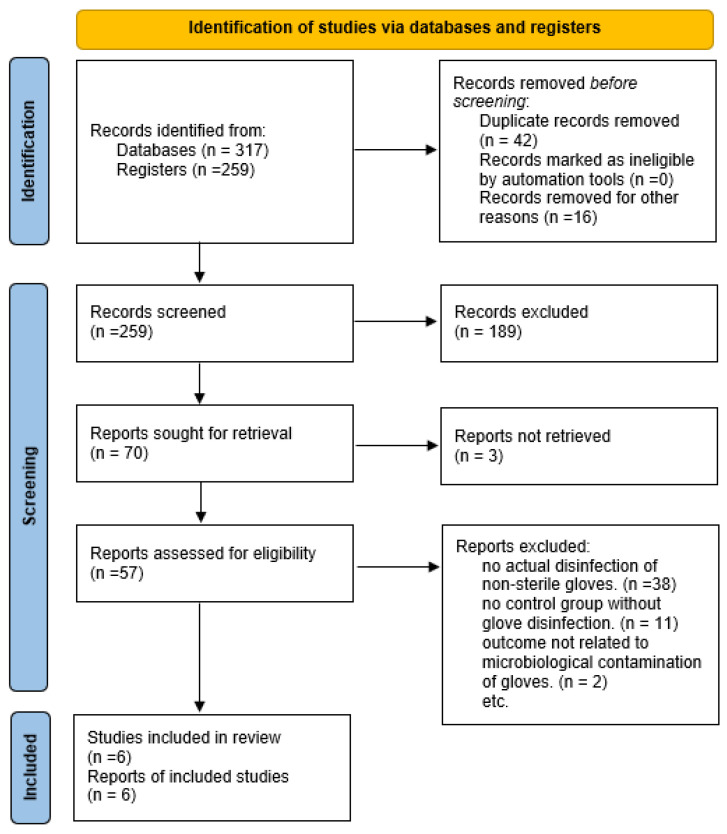
PRISMA 2020 flow diagram of study selection.

A total of 317 records were initially retrieved from the five databases (EBSCO, PubMed, Scopus, Web of Science, Ovid). After removing duplicates 259 and screening titles and abstracts, 189 records were excluded. 57 full-text studies were assessed for eligibility. Ultimately, the final analysis included a total of 6 studies (Figure 1).

### 3.2. Characteristics of Included Studies

Six studies met the inclusion criteria. Most were experimental or quasi-experimental and evaluated the microbiological contamination of non-sterile single-use gloves during patient care or PPE doffing. Sample sizes ranged from 13 to 26 healthcare workers. Interventions included alcohol-based hand rubs (ABHR), sodium hypochlorite wipes or solutions, quaternary ammonium wipes, and sporicidal ethanol. Outcomes were measured using bacterial cultures, viral surrogates, or *C. difficile* spores (Table 4).

### 3.3. Effectiveness of Glove Disinfection During PPE Doffing

Three studies assessed glove disinfection as part of PPE removal. Casanova et al. [18] demonstrated that disinfecting outer gloves before removal reduced viral transfer to inner gloves, with alcohol particularly effective against enveloped viruses, and hypochlorite showing superior activity against non-enveloped viruses. Similarly, Kpadeh-Rogers et al. [19] reported that hypochlorite and quaternary ammonium wipes reduced bacterial contamination of inner gloves by more than 99%, whereas glove change alone was insufficient to eliminate microorganisms. Tomas et al. [13] found that adding glove disinfection to standard PPE doffing training decreased contamination of hands with *C. difficile* spores from 16% to 0%, highlighting the importance of combining procedural training with chemical decontamination. Their second study confirmed that sporicidal ethanol and diluted hypochlorite were more effective against *C. difficile* spores than standard 70% ethanol.

### 3.4. Effectiveness of Glove Disinfection During Routine Patient Care

One study (Vogel et al. [20]) evaluated glove contamination during everyday patient care. After 10 min of routine tasks, 72.5% of gloves were contaminated with microorganisms. A 30 s disinfection with 70% alcohol restored sterility in 79.3% of previously contaminated gloves. These findings indicate that glove contamination occurs frequently during care, but simple alcohol-based disinfection can substantially reduce microbial load.

### 3.5. Summary of Microbiological Outcomes Across Studies

Across all studies, glove disinfection consistently reduced bacterial, viral, and spore contamination. Hypochlorite-based agents and sporicidal ethanol demonstrated the highest efficacy against spore-forming organisms such as *C. difficile*. Alcohol-based hand rubs were effective against bacteria and enveloped viruses but showed reduced activity against non-enveloped viruses and spores. Despite methodological differences between studies, the overall direction of findings was consistent.

### 3.6. Practical Implications for Clinical Use

The evidence suggests that glove disinfection—whether performed during routine patient care or as part of PPE doffing—can significantly reduce microbial contamination and lower the risk of pathogen transfer. However, no study supports using glove disinfection as a substitute for proper hand hygiene or correct PPE removal techniques. Instead, glove disinfection should be considered an adjunctive measure within broader infection-prevention strategies.

## 4. Discussion

This systematic review of six articles summarizes the available data from experimental/laboratory and clinical studies on the relationship between the disinfection of non-sterile disposable gloves and the degree of their microbial contamination during medical staff work. All included studies evaluated whether disinfection of non-sterile gloves reduces contamination of gloves and healthcare workers’ hands under various conditions, i.e., (laboratory conditions using a model virus (Φ6 and MS2), two strains of bacteria (Staphylococcus aureus MSSA and Klebsiella pneumoniae), in an infectious disease ward, and during the care of patients with CDI.

All studies showed that glove disinfection (alcohol-based preparation, wipes with hypochlorite and quaternary ammonium salts, new acidic sporicidal ethanol formulation) resulted in a significant decrease in the number of microorganisms compared to no disinfection. Nevertheless, residual contamination often remained, especially when ethanol was used and for resistant pathogens (MS2, Staphylococcus aureus MSSA, Klebsiella pneumoniae). In a study by Kpadeh-Rogers et al., [19] disinfection of outer gloves before removal reduced contamination of inner gloves by >99% when using wipes with hypochlorite or quaternary ammonium compounds (QUAT), while the use of alcohol alone did not have the same effect [19]. The superior efficacy of hypochlorite and QUAT wipes over ethanol in reducing microorganisms was confirmed by a study by Casanova L. et al. [18]. Both studies used two pairs of gloves, which is particularly important when caring for patients with VAD. In the study by Tomas et al., sporicidal ethanol and diluted hypochlorite significantly reduced the number of *C. difficile* spores on gloves, although the efficacy was lower in clinical settings than in laboratory experiments [14]. In the latest randomized clinical trial by Thom et al., the direct application of an alcohol-based hand sanitizer to non-sterile gloves worn by healthcare personnel during actual patient contacts in hospital wards was evaluated. The use of ABHR on gloves significantly reduced microbial contamination compared to usual hand hygiene practices (without instructions), but still had higher contamination levels than full compliance with the standard (glove change and hand hygiene according to the “gold standard”). In addition, rare micro-leaks in gloves were reported. These results indicate that alcohol disinfection of gloves may be a compromise between reducing contamination and the feasibility of the procedure, but it does not fully replace glove removal and hand hygiene [21].

The use of double gloves as an additional protective barrier is also important. Both the Casanova et al. and Kpadeh-Rogers et al. studies show that the second layer of gloves (inner gloves) is a key barrier: without disinfection, contamination from the outer gloves still transfers to the inner layer, albeit to a lesser extent, while disinfection before removing the outer gloves reduces contamination of the inner gloves to a very low level (>99% reduction) [18,19]. This leads to the conclusion that disinfection of outer gloves can significantly reduce the risk of transferring contamination to inner gloves, provided that hand hygiene is consistently and obligatorily performed after the gloves have been completely removed.

The most optimistic results of glove disinfection were obtained in studies conducted under strictly controlled laboratory conditions. The use of wipes saturated with sodium hypochlorite and quaternary ammonium compounds reduced the number of bacteria on the surface of gloves by more than 99% compared to simply changing gloves without disinfection [14,19,20]. In contrast, studies conducted in a real clinical setting using the same or similar agents did not show such a large reduction, although the effect was still significant [14,20]. In a study by Tomas et al., the use of hypochlorite wipes to disinfect gloves before removing PPE reduced the percentage of positive *C. difficile* cultures from the hands of staff after caring for patients with CDI [13]. Vogel A. et al., in turn, showed that after 10 min of care (T10), at least one glove was contaminated in 72.5% of care episodes (29/40), while after 30 s of disinfection with an alcohol-based preparation (T10A), both gloves were sterile in 79.3% of these 29 previously contaminated cases (23/29) [20].

This means that, under clinical conditions, disinfecting gloves with alcohol removed detectable bacterial flora from most (approximately four out of five) previously contaminated pairs of gloves, but not in all cases, confirming that real-world care conditions are more demanding than laboratory testing, although the reduction in contamination achieved remains clinically significant. In the study by Thom et al., the use of an alcohol-based hand disinfectant directly on gloves significantly reduced their contamination compared to typical clinical practice, but was not as effective as the procedure of removing gloves, hand hygiene, and putting on a new pair, which emphasizes that glove disinfection should be treated as a complementary strategy rather than a substitute for standard hand hygiene [21]. Future studies should include RCTs on MDR bacteria and longitudinal glove integrity.

## 5. Limitations

A significant limitation of the available studies is that most of them focus on selected pathogens (*C. difficile*, individual strains of MSSA and K. pneumoniae, model viruses), while in everyday clinical practice, staff are faced with frequent colonizations with multidrug-resistant strains such as carbapenemase-producing Klebsiella (e.g., MBL), vancomycin-resistant Enterococcus faecium/faecalis (VRE), various ESBL strains, and carbapenem-resistant Acinetobacter baumannii (CRAB) [23]. The risk of transmission of these microorganisms is particularly high in healthcare facilities with a limited number of sanitary facilities and multi-bed rooms. Perhaps the results of the study by Thom et al. will provide an answer to this problem, as they are the most promising of the remaining options, but still insufficient to be fully applied in clinical practice as a standard. This study clearly indicates that there is still no better solution than the gold standard of hygiene, but at the same time it gives grounds to believe that the use of ABHR on gloves may be a second-choice alternative strategy, better than no disinfection at all, but weaker than replacing gloves with hand hygiene [21]. In order to conclusively determine that the use of glove disinfection in everyday clinical practice can effectively limit the spread of multidrug-resistant strains in healthcare facilities, further research is needed to evaluate the effectiveness of this method against these pathogens. In addition, as indicated by Thom et al., due to isolated cases of microleakage after repeated disinfection, further research is needed on the effect of alcohol-based preparations on the integrity of glove material before this method is widely implemented in routine practice [21].

## 6. Conclusions

The conclusions from studies conducted in both laboratory and clinical conditions clearly emphasize the key role of hand hygiene after removing gloves, even when using multiple layers of protection, while also indicating that glove disinfection can be a useful supplement to protection against particularly virulent pathogens (EVD, CDI). Given the differences in the reduction of bacterial and viral flora from the surface of gloves depending on the agent used, it is important to select a preparation appropriate to the clinical situation and the type of pathogen. The use of an alcohol-based preparation for glove disinfection was well accepted by staff, while the use of an acidic sporicidal ethanol formulation requires caution due to its low pH, cost, organizational issues, and the need for further research on safety and efficacy. The use of sodium hypochlorite-soaked wipes in everyday practice is limited by the unpleasant smell of bleach reported by staff, concerns about respiratory tract irritation, and the risk of staining clothing [14].

## 7. Implication for Practice

The analysis of the studies included in the review clearly shows that disinfection of non-sterile gloves can significantly reduce the microbial contamination of hands and surfaces, especially when caring for patients with CDI or other highly infectious pathogens [13]. The use of double gloves with disinfection of the outer layer during doffing significantly reduces the transmission of microorganisms to the inner gloves (>99% reduction in laboratory studies), which may be particularly useful when caring for patients with EVD or other high-risk pathogens. It is crucial to select a disinfectant appropriate for the type of pathogen, as the effectiveness of alcohol, hypochlorite, and sporicidal preparations varies significantly. The results of these studies clearly indicate the place of glove disinfection in clinical practice: it is only a supplement to, not a replacement for, the gold standard of hand hygiene. Furthermore, glove disinfection can be considered a “second choice” strategy in situations where full compliance with the standard (glove change and hand hygiene) is temporarily or organizationally unfeasible.

## Figures and Tables

**Table 1:** PICO framework, inclusion and exclusion criteria.

	Inclusion Criteria	Exclusion Criteria
Population (P)	Medical personnel: studies on medical personnel using non-sterile gloves in laboratory and hospital settings	E.g., research involving non-medical personnel (e.g., cleaning, administrative, technical staff). Research involving only medical students.
Intervention (I)	Disinfection of disposable gloves	Disinfection of other PPE, e.g., face shields, chest areas of protective suits, and soles of shoes/shoe covers.
Comparison (C)	No disinfection of gloves	n/a
Outcome (O)	microbiological contamination of glove surfaces	Other outcome (e.g., studies were excluded where the primary endpoint was only material integrity/glove wear (e.g., perforations, changes in strength after repeated disinfection), without assessment of microbial contamination)
Study Type	RCTs, experimental simulation studies, quasi-experimental before–after study, prospective observational study	Letters to the editor Case study report Quantitative studies Qualitative studies Reviews (any types)
Years considered/Time period	2015–2025	prior to 2015
Language	English	Other languages
Databases	EBSCO, PubMed, Scopus, Web of Science, and Ovid	Other databases, Gray literature,

n/a—not applicable.

**Table 2:** Search strategy, filters and results.

Database	Search Strategy	Filters	Results
EBSCO	(Health Personnel OR “healthcare workers” OR “medical staff” OR nurses) AND (disinfection OR decontamination OR sanitization OR “hand gloves disinfection”) AND (“microbial contamination” OR contamination OR “bacterial contamination”)	-Medline, CINAHL ultimate, -years: 2015–2025, -English	249
PubMed	(Health Personnel[Mesh] OR “healthcare workers”[tw]) AND (Gloves[Mesh] OR gloves[tw] OR “disposable gloves”[tw]) AND (Disinfection[Mesh] OR disinfection[tw] OR decontamination[tw] OR sanitization[tw])	-randomized controlled trial, clinical trial -years: 2015–2025, -English	7
Scopus	TITLE-ABS-KEY (“health personnel” OR “healthcare workers” OR “medical staff” OR nurses) AND TITLE-ABS-KEY (gloves OR “disposable gloves”) AND TITLE-ABS-KEY (disinfection OR decontamination OR sanitization) AND TITLE-ABS-KEY (“microbial contamination” OR contamination OR “bacterial contamination”) AND PUBYEAR > 2014 AND PUBYEAR < 2025 AND (LIMIT-TO (DOCTYPE, “ar”)) AND (LIMIT-TO (LANGUAGE, “English”))	-articles, reviews, -years: 2015–2025, -English	36
Web of Science	(“healthcare worker” OR “medical staff” OR nurse OR doctor OR “medical personnel”) AND (glove OR “single-use glove”) AND (disinfection OR decontamination OR sterilization OR cleaning) AND (contamination OR “microbiological contamination” OR “bacterial contamination”)	-articles, reviews, -years: 2015–2025, -English	19
Ovid	exp Health Personnel/or “healthcare workers”.tw. or “medical staff”.tw. AND exp Gloves, Protective/or gloves.tw. or “disposable gloves”.tw. or “non-sterile gloves”.tw. AND exp Disinfection/or disinfection.tw. or decontamination.tw. or sanitization.tw. or cleaning.tw.	-randomized controlled trial, clinical trial, -years: 2015–2025, -English	6

**Table 3:** JBI Critical Appraisal Tools—checklist for randomized controlled trials.

Author, Year	Q1	Q2	Q3	Q4	Q5	Q6	Q7	Q8	Q9	Q10	Q11	Q12	Q13	Quality Appraisal
Casanova L. et al., 2016 [18]	U	U	Y	Y	Y	Y	Y	Y	Y	Y	Y	Y	N	10/13 (moderate quality)
Kpadeh-Rogers Z. et al., 2019 [19]	U	U	Y	N	Y	Y	Y	U	Y	Y	Y	Y	N	8/13 (moderate quality)
Tomas M E. et al., 2015 [13]	Y	N	Y	U	Y	Y	Y	N	Y	Y	Y	Y	Y	10/13 (moderate quality)
Tomas M E. et al., 2016 [14]	N	U	Y	N	Y	Y	Y	Y	Y	Y	Y	Y	Y	10/13 (moderate quality)
Vogel A. et al., 2021 [20]	U	N	Y	N	Y	Y	Y	N	U	Y	Y	Y	N	7/13 (moderate quality)
Thom K. A. et al., 2024 [21]	Y	U	Y	U	Y	Y	U	Y	Y	Y	Y	Y	N	9/13 (moderate quality)

Y—Yes, N—No, U—unclear. Q1—Was true randomization used for assignment of participants to treatment groups? Q2—Was allocation to treatment groups concealed? Q3—Were treatment groups similar at the baseline? Q4—Were participants blind to treatment assignment? Q5—Were those delivering treatment blind to treatment assignment? Q6—Were outcomes assessors blind to treatment assignment? Q7—Were treatment groups treated identically other than the intervention of interest? Q8—Was follow up complete and if not, were differences between groups in terms of their follow up adequately described and analyzed? Q9—Were participants analyzed in the groups to which they were randomized? Q10—Were outcomes measured in the same way for treatment groups? Q11—Were outcomes measured in a reliable way? Q12—Was appropriate statistical analysis used? Q13—Was the trial design appropriate, and any deviations from the standard RCT design (individual randomization, parallel groups) accounted for in the conduct and analysis of the trial?

**Table 4:** Summary of studies on disinfection of non-sterile single-use gloves.

Author, Year/Country	Design, Setting, Sample	Intervention (Disinfection Method)	Main Microbiological Outcomes	Implications for Practice
Casanova et al., 2016, USA [18]	Experimental simulation; 15 HCWs	Double gloves; disinfection of outer gloves with ABHR or sodium hypochlorite	Alcohol eliminated enveloped virus; hypochlorite more effective for non-enveloped virus; occasional transfer remained possible	Disinfection during doffing reduces viral transfer but does not replace hand hygiene
Kpadeh-Rogers et al., 2019, USA [19]	Laboratory simulation; 20 HCWs	Outer gloves contaminated with MSSA and *K. pneumoniae*; ABHR, hypochlorite wipes, QUAT wipes vs. glove change only	Hypochlorite and QUAT achieved >99% bacterial reduction; glove change alone insufficient	Glove disinfection significantly lowers bacterial load, but hand hygiene remains essential
Tomas et al., 2015, USA [13]	Quasi-experimental; infectious diseases ward; 26 HCWs	PPE doffing training + glove disinfection with hypochlorite wipes	*C. difficile* contamination on hands decreased from 16% to 0%	Combining doffing training with glove disinfection prevents detectable spore contamination
Tomas et al., 2016, USA [14]	Laboratory + clinical; HCWs caring for CDI patients	Sporicidal acidic ethanol, diluted hypochlorite vs. 70% ethanol	Sporicidal ethanol and diluted hypochlorite most effective against spores; 70% ethanol least effective	Sporicidal ethanol may aid CDI prevention but requires further evaluation
Vogel et al., 2021, France [20]	Experimental study; 13 HCWs; 40 care episodes	Gloves disinfected with 70% alcohol for 30 s during patient care	72.5% of gloves contaminated after 10 min; 79.3% became sterile after disinfection	Alcohol-based disinfection greatly reduces contamination during routine care
Thom K. A. et al., 2024, USA [21]	3-arm RCT; adult & pediatric med-surg/intermediate/ICU; HCP caring for patients on contact precautions	ABHR applied to gloved hands vs. gold standard (glove removal + HH + new gloves) vs. usual care	ABHR reduced glove contamination vs. usual care, but was less effective than gold standard; rare microperforations.	ABHR on gloves is faster and improves glove decontamination compared with usual care but does not replace glove change and HH

Note. Most included studies were experimental or quasi-experimental with relatively small samples and controlled contamination models; therefore, results should be interpreted with caution. Abbreviations: ABHR—alcohol-based hand rub; HCW—healthcare worker; CDI—Clostridioides difficile infection; PPE—personal protective equipment; QUAT—quaternary ammonium compound; MSSA—Methicillin-sensitive Staphylococcus aureus. Overall conclusion. Glove disinfection reliably reduced microbial contamination but cannot replace proper hand hygiene.

## Data Availability

No new data were created or analyzed in this study. Data sharing is not applicable to this article.

## Supplementary Materials

The following supporting information can be downloaded at: https://www.mdpi.com/article/10.3390/microorganisms14020286/s1.
